# Integrative single-cell RNA sequencing and metabolomics decipher the imbalanced lipid-metabolism in maladaptive immune responses during sepsis

**DOI:** 10.3389/fimmu.2023.1181697

**Published:** 2023-04-27

**Authors:** Han She, Lei Tan, Yi Wang, Yuanlin Du, Yuanqun Zhou, Jun Zhang, Yunxia Du, Ningke Guo, Zhengbin Wu, Qinghui Li, Daiqin Bao, Qingxiang Mao, Yi Hu, Liangming Liu, Tao Li

**Affiliations:** ^1^ State Key Laboratory of Trauma, Burns and Combined Injury, Shock and Transfusion Department, Daping Hospital, Army Medical University, Chongqing, China; ^2^ Department of Anesthesiology, Daping Hospital, Army Medical University, Chongqing, China; ^3^ Department of Intensive Care Unit, Daping Hospital, Army Medical University, Chongqing, China

**Keywords:** sepsis, lipid-metabolism, machine learning algorithm, single-cell RNA sequencing, metabolomics

## Abstract

**Background:**

To identify differentially expressed lipid metabolism-related genes (DE-LMRGs) responsible for immune dysfunction in sepsis.

**Methods:**

The lipid metabolism-related hub genes were screened using machine learning algorithms, and the immune cell infiltration of these hub genes were assessed by CIBERSORT and Single-sample GSEA. Next, the immune function of these hub genes at the single-cell level were validated by comparing multiregional immune landscapes between septic patients (SP) and healthy control (HC). Then, the support vector machine-recursive feature elimination (SVM-RFE) algorithm was conducted to compare the significantly altered metabolites critical to hub genes between SP and HC. Furthermore, the role of the key hub gene was verified in sepsis rats and LPS-induced cardiomyocytes, respectively.

**Results:**

A total of 508 DE-LMRGs were identified between SP and HC, and 5 hub genes relevant to lipid metabolism (*MAPK14, EPHX2, BMX, FCER1A*, and *PAFAH2*) were screened. Then, we found an immunosuppressive microenvironment in sepsis. The role of hub genes in immune cells was further confirmed by the single-cell RNA landscape. Moreover, significantly altered metabolites were mainly enriched in lipid metabolism-related signaling pathways and were associated with *MAPK14.* Finally, inhibiting *MAPK14* decreased the levels of inflammatory cytokines and improved the survival and myocardial injury of sepsis.

**Conclusion:**

The lipid metabolism-related hub genes may have great potential in prognosis prediction and precise treatment for sepsis patients.

## Introduction

Sepsis can advance to fatal organ failure which is caused by uncontrolled immune response to infection and associated with high morbidity and mortality (1, 2), despite evolving concepts and developments in multi-disciplinary approaches (3). Early diagnosis in order to early intervention before organ dysfunction is critical to improving survival rate of sepsis (4). Due to their poor sensitivity and specificity in assessing the substantial disease heterogeneity, the existing biological markers cannot be used for sepsis prognosis prediction (5–8). Therefore, further studies are required to investigate the pathogenesis of sepsis and identify more sensitive and specific therapeutic targets.

Metabolic and immune reactions often occur in the early stage of sepsis without intense histologic changes, and can reflect the severity of later organ failure (9–11). Lipid metabolic changes and the activation of lipid-related pathways are important features underlying the pathophysiology of sepsis (12). However, the underlying metabolites and their association with lipid metabolism-related genes in septic patients remain unidentified. A better understanding of the alterations in lipid metabolism and immune cell infiltration could contribute to identifying more potential therapeutic targets to modulate lipid metabolism during sepsis pharmacologically. In recent years, the application of machine learning has received widespread attention and recognition due to its ability to promote personalized medicine and assist computer-aided diagnosis (13). Besides, the emerging ‘Omic’ technologies can provided more comprehensive knowledge about the whole picture of immune cell profiles in sepsis (14, 15). Metabolomics, which integrate genomics, transcriptomics, and proteomics based on the omics technology, exhibit huge advantages in exploring biology (16, 17). Thus, we used machine learning algorithms for screening lipid metabolism-related hub genes, and characterized them with metabolomic profiling (18).

The activation of immune cells in reaction to the pathogens accountable for the onset of sepsis is regulated by immunometabolism, which is the metabolic stage of the immune cells. An alteration in the immunometabolism can trigger the disturbance of immune response during sepsis (19). To explore the molecular mechanisms underlying sepsis, previous studies mainly characterized changes in gene expression profiles and relevant cellular pathways (20, 21). Although sufficient information has been obtained for analysis, studies that sequence pooled populations of immune cells, rather than each individual cell, enlarge the cellular heterogeneity and probably confound the interpretation of the immune response. Thanks to the technological advances, gene expression analysis can be performed at a higher resolution, and single-cell RNA sequencing enables the determination of the precise gene expression patterns at the single-cell level (22). However, changes in transcriptional states of immune cell-type specific signatures during sepsis are miscellaneous and largely unknown. Thus, we characterized the spectrum of immune cell states of sepsis patients by single-cell-resolved gene expression profiling.

In the present study, we identified novel lipid metabolism-related hub genes in sepsis *via* machine learning algorithms, and investigated the roles of these hub genes in immune cell infiltration features by single-cell RNA-seq analysis. Metabolomics was used to determine the most relevant metabolites with these hub genes. We aimed to discover the possible treatment intervention for sepsis.

## Materials and methods

### Reagents

SB203580 (Cat. HY-10256) was purchased from MedChemExpress (Monmouth, NJ, America). Lipopolysaccharide was purchased from Sigma (Cat. L4130, St. Louis, MO, America). Antibodies for MAPK14 (Cat. 8690S) and β-actin (Cat. 4970S) were purchased from Cell Signaling Technology (Danvers, Massachusetts, America). Cell counting kit-8 (Cat. C0038) was purchased from Beyotime Biotechnology (Shanghai, China). Enzyme-linked immunosorbent assay (ELISA) detection kit of pro-inflammatory cytokines TNF-α (E-EL-R2856c), interleukin (IL)-6 (E-EL-R0015c), and IL-1β (E-EL-R0012c) were purchased from Elabscience (Wuhan, China). *In situ* cell death detection kit (Cat. 11684795910) was purchased from Roche (Huntsville, German).

### Study design and population recruitment

A total of 30 sepsis patients diagnosed according to the Sepsis-3 criteria were recruited from Daping Hospital, along with 15 age-matched healthy volunteers (the inclusion and exclusion criteria for sepsis patients are presented in **Supplementary Table 1**). The study received approval from the Ethics Committee and was registered with the Chinese Clinical Trial Registry (ChiCTR2200055772). The healthy controls did not take any medications and had no comorbidities. All participants were admitted between December 2021 and April 2022 and provided written informed consent prior to inclusion in the study. Blood samples were collected from all participants within 24 hours of admission or enrollment, and the serum was subsequently isolated and stored at −80°C for further analysis.

### Dataset collection

The mRNA matrix for this study was obtained from the Gene Expression Omnibus database (https://www.ncbi.nlm.nih.gov/geo/). Three datasets were collected for subsequent analysis, including GSE65682, GSE95233, and GSE54514. GSE65682, which contains 760 sepsis patients and 42 healthy controls, was used as the training cohort for machine learning. GSE95233 (consisting of 51 sepsis patients and 22 healthy controls) and GSE54514 (comprising 35 sepsis patients and 18 healthy controls) were merged as a validation cohort to verify the mRNA expression and diagnostic performance of hub genes. Lipid metabolism-related genes (LMRGs) were obtained from the KEGG, Reactome, and Uniprot databases by searching for the term “lipid metabolism,” resulting in a total of 1079 LMRGs for investigation. The analysis of differentially expressed LMRGs between sepsis patients (SP) and healthy controls (HC) was conducted using the “limma” R package, with a threshold set at a P-value < 0.05.

### Sepsis model establishment

Animal experiments in this study were conducted in accordance with the Animal Research: Reporting of *In Vivo* Experiments (ARRIVE) guidelines. Adult Sprague-Dawley rats weighing 200-220g (n=192) were bred in the animal facility and provided with ad libitum food and water. The rats were randomly divided into three groups: the control group, the sepsis group, and the SB203580-treated sepsis group. To establish a sepsis model, cecal ligation and puncture (CLP) were performed as previously described (23). SB203580 (500 μg/kg) was administered *via* the tail vein 30 minutes prior to sepsis induction, while the sepsis group received an equal volume of ddH2O in the same manner. Myocardial tissues and peripheral blood were collected 12 hours after CLP.

### Gene co-expression networks of DE-LMRGs

To screen co-expression networks of differentially expressed lipid metabolism-related genes (DE-LMRGs) in sepsis, the weighted gene co-expression network analysis (WGCNA) algorithm implemented in the R package was employed. The appropriate power index of β was selected using the criterion of scale-free topology with an R^2^ cutoff of 0.85. The adjacency matrix was then transformed into a topological overlap matrix, and average linkage hierarchical clustering was applied to classify all DEGs with similar expression profiles into different modules. The most central genes in these modules were further identified as hub genes.

### Identification of DE-LMRGs *via* LASSO and random forest algorithm

To identify diagnostic feature biomarkers, this study applied multiple machine learning algorithms. First, LASSO logistic regression was performed with ten-fold cross-validation to screen candidate iteratively reweighted least square. The algorithm was run for 1000 cycles to select feature variables based on 1-se criteria or minimum criteria. Next, the RF algorithm based on classification and regression tree was applied, with the expression matrix of all genes as features and disease state as a label. A Venn diagram was used to identify the common hub genes among RF, LASSO, and WGCNA. The different expressions of these hub genes were analyzed between SP and HC in the training and validation datasets, respectively. Finally, the classification performance of the hub genes in both the training and validation cohorts was assessed using the receiver operating characteristic (ROC) curve, and the area under the curve was calculated.

### Immune infiltration analysis

To determine the proportions of immune cells, this study applied the CIBERSORT and ssGSEA algorithms. Correlation analysis was then performed to analyze the association between immune cells and LMRGs.

### Single cell RNA-seq analysis

The scRNA-seq dataset GSE167363 (24) (included 5 sepsis patients and 2 healthy controls) was analyzed in this study. Quality control was performed, and expression matrix files were generated based on gene counts and UMI counts. Cells were filtered based on gene counts between 200 to 5,000 and UMI counts below 30,000, and a total of 38,562 cells were retained for downstream analysis. Seurat v3.1.2 (25) was used for dimension reduction and clustering, and Harmony was used for batch correction. The top 2000 variable genes were selected, and cells were separated into 23 clusters by the Find Clusters function. Sub-clustering analysis of cell types was performed with a resolution of 1.2. Cells were visualized in a two-dimensional space using UMAP. Cell-cell interaction (CCI) analysis between HC and SP was performed using Cellphone DB v2.1.0, based on ligand-receptor pairs. Cell differentiation trajectory was reconstructed with Monocle2 (26), and DDRTree was used for FindVariableFeatures and dimension reduction. The trajectory was visualized using the plot_cell_trajectory function. DEGs were used to sort cells in order of spatial-temporal differentiation.

### Metabolomics profiling

Metabolomics analysis was conducted on peripheral blood specimens using a UHPLC system (Vanquish, Thermo Fisher Scientific). The MS/MS spectra were acquired by the Orbitrap Exploris 120 mass spectrometer (Xcalibur, Thermo) on information-dependent acquisition (IDA) mode under the control of the acquisition software. Filter individual peaks to remove noise. Filter deviation values based on relative standard deviation (RSD), which is the coefficient of variation (CV). Afterwards, simulate missing value recoding in the original data. The numerical simulation method fills in half of the minimum value. The resulting three-dimensional data, which included peak number, sample name, and normalized peak area, were input into the SIMCA14+ metaboanalyst tool package (Umetrics, Umea, Sweden) for PCA analysis (27). The significantly different expressions of metabolites and metabolic pathways were analyzed by heatmap and bubble plot. The significantly different expressions of metabolites and metabolic pathways were analyzed using a heatmap and bubble plot. Subsequently, a support vector machine was used to develop a classifier to verify the key metabolites that can distinguish sepsis patients from healthy controls. The metabolomics profiling data for this study has been deposited into the CNGB Sequence Archive (CNSA) of the China National GeneBank DataBase (CNGBdb) (https://db.cngb.org/data_access/) with the accession number CNP0004111.

### Agarose gel electrophoresis of reverse transcriptase-polymerase chain reaction

RNA was extracted from human blood samples and rat cardiac tissues as previously described (23). Following reverse transcription, the resulting products in each experimental group were subjected to PCR amplification. The PCR products were then separated by electrophoresis and visualized under ultraviolet light. The primers utilized in these experiments are listed in **Supplementary Table 2**.

### Cell culture and treatment

The H9C2 cells were cultured in DMEM (Invitrogen, CA, USA) supplemented with 10% fetal bovine serum (v/v) (FBS; Gibco, MD, USA), 1000 U/ml penicillin, and 100 μg/ml streptomycin (Invitrogen, CA, USA). To establish a sepsis model, cells were stimulated with 500 ng/ml LPS for 12 hours. Cells in the normal group were incubated with an equal volume of DMEM. In the SB203580 group, cells were pre-incubated with SB203580 at a concentration of 2 μM for 30 minutes before stimulation with LPS.

### Western blotting

Cells were lysed with RIPA lysis buffer with protease inhibitor (Roche, USA). Cells were lysed using RIPA lysis buffer with a protease inhibitor (Roche, USA). The total protein concentration was quantified using a BCA protein assay kit. After separation by SDS-PAGE, the proteins were transferred onto a PVDF membrane. The membrane was then blocked with 5% bovine serum albumin for 1 hour at room temperature and incubated with primary antibodies against MAPK14 (1:1000) and β-actin (1:4000) overnight at 4°C. Subsequently, the membrane was rinsed with PBS and incubated with a goat anti-rabbit secondary antibody (1:20000) for 1 hour at room temperature. The signals were read and analyzed using Image Lab software (Bio-Rad).

### Statistical analysis

The statistical analyses were conducted using R software, version 4.1.2 (http://www.r-project.org). Pearson’s correlation was used to adjust the correlation of co-expression. A p-value less than 0.05 was considered statistically significant.

## Results

### The lipid metabolism-related hubgenes in sepsis were screened *via* various machine learning algorithms

**Figure 1:**
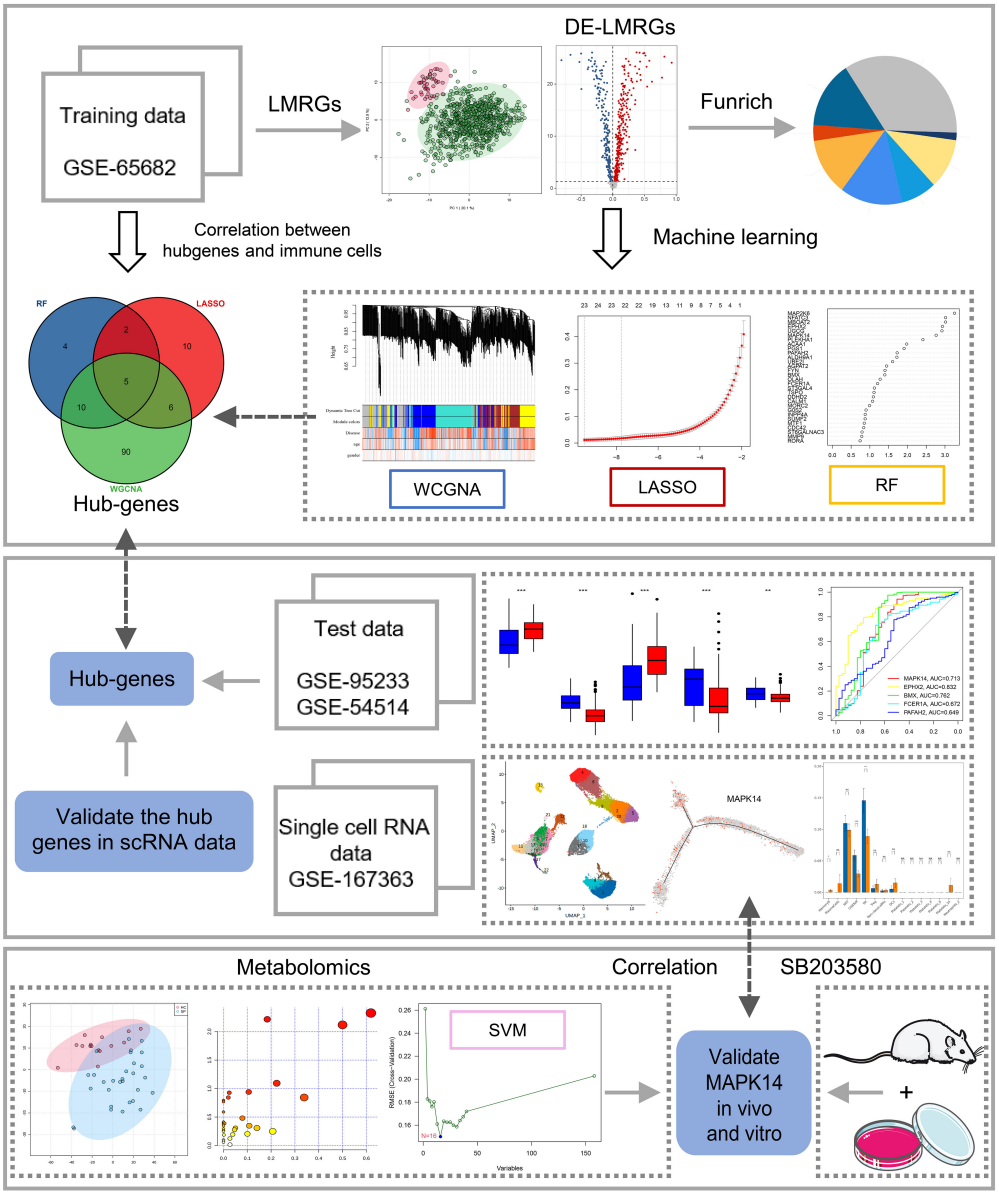
Study flowchart. The scheme diagram of data analyzing. *: as compared with the Control group, P <0.05; **: as compared with the Control group, P<0.01; ***: as compared with the Control group, P<0.001; ns: as compared with the Control group,no significant difference. NA:Not available.

**Figure 2:**
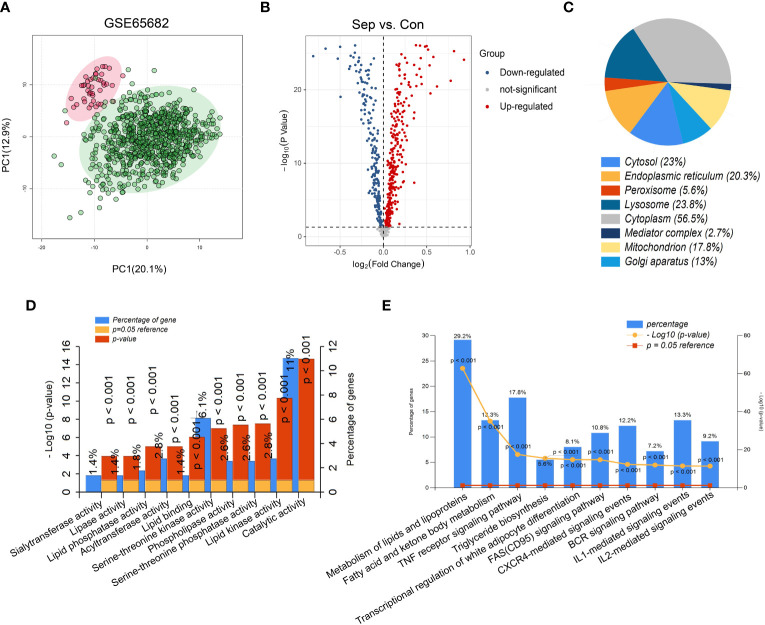
Identification of differentially expressed lipid metabolism-related genes (DE-LMRGs) from GEO dataset. **(A)** Principal Components Analysis (PCA) score plot of GSE65682. Each scatter represents a sample. The red represents the control group, and the green represents the sepsis group. **(B)** Volcano plot of DE-LMRGs in GSE65682. The blue dots indicate down-regulated DEGs while the red dots indicate up-regulated DEGs. Statistically significant DEGs were identified as those with a student’s t-test *P* < 0.05 and a fold-change > 1. Cellular compound **(C)**, Molecular function **(D)** and Biological Pathway **(E)** of DE-LMRGs analyzed by Funrich.

The flow chart of this study is shown in **Figure 1**. The results of PCA showed that gene expression patterns were significantly different between sepsis patients and healthy controls (**Figure 2A**). Compared with the healthy control group, a total of 508 differentially expressed lipid metabolism-related genes (DE-LMRGs) were involved in GSE65682, 307 of which were upregulated and 201 downregulated (fold-change > 1, P< 0.05) (**Figure 2B**). As for GO analysis, Cellular compounds (CCs) showed that 56.5% of these DE-LMRGs were distributed in the cytoplasm, 23% in the cytosol, and 20.3% in the endoplasmic reticulum (**Figure 2C**). The top 10 Molecular functions (MFs) included catalytic activity, serine-threonine kinase activity, acyltransferase activity, lipid kinase activity, etc. (**Figure 2D**). In addition, the top 10 Biological Pathways (BPs) of DE-LMRGs were shown in **Figure 2E**, including the metabolism of lipids and lipoproteins, TNF receptor signaling pathway, fatty acid, and ketone body metabolism, and IL-1-mediated signaling events.

**Figure 3:**
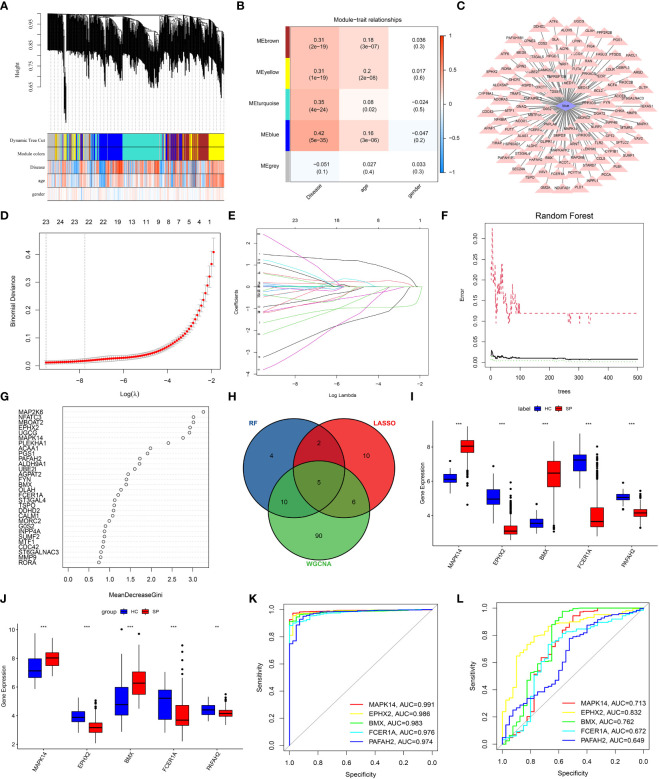
Screening of DE-LMRGs *via* the comprehensive strategy. **(A)** Clustering dendrogram of differentially expressed genes related to sepsis. **(B)** Heatmap between the correlation between modules and disease (Each cell contained the correlation coefficient and corresponding P-value). **(C)** The gene significance for sepsis in the blue module. **(D, E)** Least absolute shrinkage and selection operator (LASSO) logistic regression algorithm to screen DE-LMRGs. Different colors represent different genes. **(F, G)** Based on random forest (RF) algorithm to screen DE-LMRGs. Genes with an importance score greater than 1 were used for subsequent signature establishment. **(H)** VENN diagram of hub genes. **(I, J)** Validation of expression of hub genes in patients with sepsis and normal control in the training cohort and the validation cohort. **(K, L)** ROC of hub genes in the training cohort and the validation cohort. **: as compared with the Control group, *P <*0.01; ***: as compared with the Control group, *P*<0.001.

WGCNA was performed to find suspected modules of sepsis. A soft threshold of β=5 was chosen to ensure the network is scale-free (**Figure 3A**). The expression matrix was transformed into an adjacency matrix and converted into a topological matrix. Genes were then hierarchically clustered and visualized in a dendrogram according to the dissimilarity topological overlap matrix. The module eigengenes (MEs) were determined as the first principal component of each gene module. We then sought correlations between MEs with disease, age, and gender to determine sepsis-associated modules. The blue module had the highest correlation with sepsis (r = 0.42, P = 5e -35) (**Figure 3B**). The genes in the blue module were shown (**Figure 3C**).

LASSO logistic regression was established to shrink the regression coefficients towards zero and select out DE-LMRGs. As shown in **Figures 3D, E**, a total of 23 DE-LMRGs were screened. Likewise, RF was also built with minimum error regression trees for DE-LMRGs screening (**Figures 3F, G**), and 21 DE-LMRGs were screened.

By intersection of these 3 methods (WGCNA, LASSO, and RF), 5 hub genes (*MAPK14*, *EPHX2*, *BMX*, *FCER1A*, and *PAFAH2*) were determined (**Figure 3H**). Then, the expressions of these 5 hub genes were validated in the training cohort (GSE65682) and the merged validation cohort (GSE95233 and GSE54514), respectively (**Figures 3I, J**). ROC curves showed that these hub genes had an excellent prediction ability for sepsis in the training cohort GSE65682 with the AUC ratio>90% (**Figure 3K**). In the merged validation cohort, the prediction ability of *MAPK 14, EPHX2, BMX, FCER1A*, and *PAFAH2* were validated with an AUC of 0.713, 0.832, 0.762, 0.672, and 0.649, respectively (**Figure 3L**).

### The lipid metabolism-related hubgenes could affect the immune cell infiltration in sepsis

**Figure 4:**
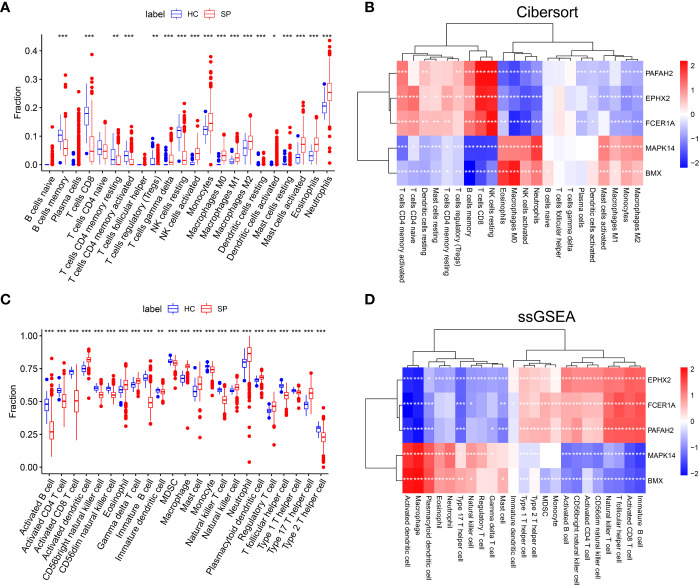
The landscape of Immune cell infiltration and correlation analysis in GSE65682. **(A)** Analysis of immune-cell proportion comparisons between sepsis patients and healthy controls by CIBERSORT. **(B)** Person’s correlation analysis of CIBERSORT between infiltrating immune cells and identified hub genes. Red nodes indicate positive correlation while blue nodes indicate negative correlation. **(C)** Analysis of immune-cell proportion comparisons between sepsis patients and healthy controls (The blue and red boxplots stand for control and sepsis, respectively) by ssGSEA. **(D)** Person’s correlation analysis of ssGSEA between infiltrating immune cells and identified hub genes. Red nodes indicate positive correlation while blue nodes indicate negative correlation. *: as compared with the Control group, *P <*0.05; **: as compared with the Control group, *P*<0.01; ***: as compared with the Control group, *P*<0.001.

The immune-cell infiltration between SP and HC was analyzed. First, immune-cell proportion comparisons were analyzed by CIBERSORT in each sample of the training dataset GSE65682 (**Figure 4A**). Next, as the Pearson’s showed (**Figure 4B**), there was a positive correlation between 3 hub genes (*PAFAH2, EPHX2, FCER1A*) and CD4^+^ T cells, CD8^+^ T cells, resting NK cells, and regulatory T cells. In contrast, a positive correlation was found between the other 2 hub genes (*MAPK14* and *BMX*) and M1 macrophages, M2 macrophages, monocytes, and activated mast cells. The above results were also validated by the ssGSEA algorithm (**Figures 4C, D**), suggesting an immunosuppressive microenvironment in sepsis, which might provide novel strategies for immunotherapy.

### MAPK14 was involved in the differentiation of monocytes in sepsis

**Figure 5:**
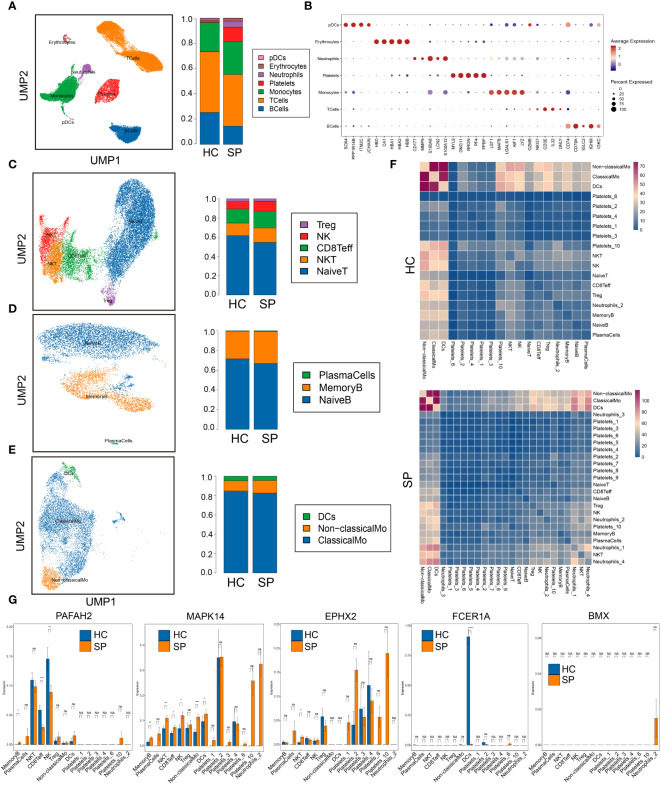
Single-cell gene expression analysis of peripheral blood mononuclear cells (PBMC) in sepsis. **(A)** UMAP plot of the cell clusters annotated by the SingleR package. **(B)** Bubble chart of the top five markers of each cluster. **(C)** Subclusters of T cells. **(D)** Subclusters of B cells. **(E)** Subclusters of monocytes. **(F)** Heatmaps of number of ligand-receptor pairs across cell subgroups in HC and SP. **(G)** The expressions of the 5 hub genes in each subclusters. *: as compared with the Control group, *P <*0.05; **: as compared with the Control group, *P <*0.01; ***: as compared with the Control group, *P*<0.001; ****: as compared with the Control group, *P*<0.0001. ns: as compared with the Control group, no significant difference. NA: Not available.

To further investigate the immune cell landscape of these hub genes at the single cell level, we downloaded scRNA-seq data from GSE167363 (HC=2, SP=5) to explore subpopulations in sepsis. The samples in this data were peripheral blood mononuclear cells (PBMCs). We obtained 38562 high-quality single-cell data after the quality control. Then normalization, unsupervised dimensionality reduction, and graph-based clustering were performed, and the cell type of each cluster was determined with the expression of canonical markers found in the DEGs using the SynEcoSys database. Finally, 7 cell clusters (plasmacytoid dendritic cells, erythrocytes, neutrophils, platelets, monocytes, T cells, and B cells) were obtained in the UMAP plot, which distributed unevenly between HC and SP (**Figure 5A**). The top 5 markers of each cluster were visualized in the bubble chart (**Figure 5B**). The clusters in each sample were displayed separately in UMAP plots (**Supplementary Figure 1A**). The top 3 ranked cell populations were T cells, B cells, and monocytes in both HC and SP. DEGs between HC and SP in T cells, B cells, and monocytes were shown respectively in **Supplementary Figure 1B**.

To identify subtypes of immune cells in sepsis, we clustered T cells, B cells, and monocytes, respectively. Five subsets of T cells were identified, including Treg, NK, CD8^+^ Teff, NK T cells, and naive T cells, and they were distributed unevenly between SP and HC (**Figure 5C**). Three subsets of B cells were identified, including plasma cells, memory B cells, and naive B cells (**Figure 5D**). Three subsets of monocytes were identified, including DCs, non-classical monocytes, and classical monocytes, and the fraction of non-classical monocytes was higher in SP than that in HC (**Figure 5E**). In addition, platelets were subclustered into 10 subsets (**Supplementary Figure 2A**). Neutrophils were subclustered into 4 sub-populations in SP, while only one sub-population in HC (**Supplementary Figure 2B**). From the heatmaps of the ligand-receptor pairs across cell subgroups from HC (upper panel) and SP (lower panel) (**Figure 5F**), we found that the monocytes population (DCs, non-classical monocytes, and classical monocytes) harbored the maximum number of cell-cell crosstalk with other neighboring cells both in SP and in HC. Then, the expressions of 5 hub genes (*PAFAH2*, *MAPK14*, *EPHX2*, *FCER1A*, and *BMX*) in each subpopulation were shown respectively in **Figure 5G**. All these hub genes showed significant differences between SP and HC, indicating that lipid metabolism-related genes may play key roles in most immune cell subclusters during sepsis.

**Figure 6:**
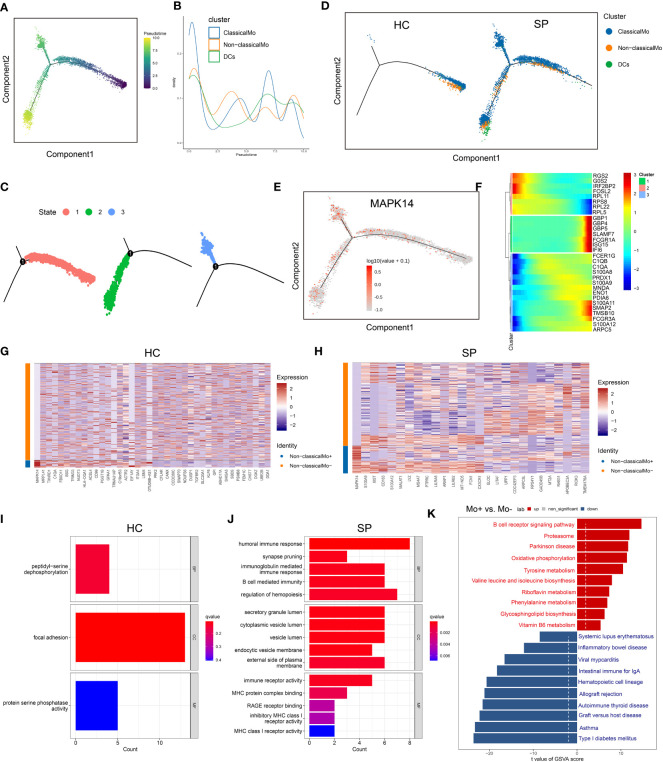
Trajectory and pseudo-time analysis of immune cells. **(A)** Monocle pseudotime trajectory of monocytes. **(B)** Curve plot showing the progression of classical monocytes, non-classical monocytes and DCs. **(C)** The pseudotime trajectory revealed 3 different states of monocytes. **(D)** Monocle pseudotime trajectory showing the progression of classical monocytes, non-classical monocytes and DCs in HC and SP. **(E)** Monocle pseudotime trajectory of MAPK14. **(F)** The clustering and expression kinetics shows top 30 representative DEGs along the main stem of the pseudotime trajectory. **(G)** Heatmap showing the top 40 DEGs between non-classical monocytes and non-classical mo^-^ cells in HC. **(H)** Heatmap showing the top 40 DEGs between non-classical monocytes and non-classical mo^-^ cells in SP. **(I)** Bar graph of GO Enrichment analysis in HC. **(J)** Bar graph of GO Enrichment analysis in SP. **(K)** Bar plot of GSVA analysis (Mo+ *vs* Mo-).

Because monocytes played an essential role in cell communication, we focused further investigated cell differentiation by trajectory and pseudotime analysis *via* Monocle. The classical monocytes were first developed into non-classical monocytes, which further developed into the DCs. In SP, all the 3 states of cells could be detected, while only state 1 was found in HC (**Figures 6A–D**). Interestingly, one of the hub genes, MAPK14, was simultaneously present in all the states (**Figure 6E**). DEGs were identified along the main stem of the pseudotime trajectory, and the top 30 representative DEGs were shown in the clustering and expression kinetics (**Figure 6F**). Cells in clusters expressing *MAPK14* were labeled “positive”; otherwise, cells were labeled “negative”. In addition, the top 40 DEGs between non-classical monocytes (positive) and non-classical mo^-^(negative) cells in HC and SP were exhibited in the heatmaps (**Figures 6G, H**). Based on GO analysis, the biological processes of DEGs between non-classical monocytes and non-classical mo^-^ cells in SP were identified as humoral immune response, synapse pruning, and immunoglobulin-mediated immune response (**Figures 6I, J**). By GSVA analysis, we detected that the up-regulated pathways in non-classical monocytes (positive) were the B cell receptor signaling pathway, oxidative phosphorylation and so on (**Figure 6K**).

### The role of MAPK14 in sepsis patients, rats and LPS-induced cardiomyocytes

**Figure 7:**
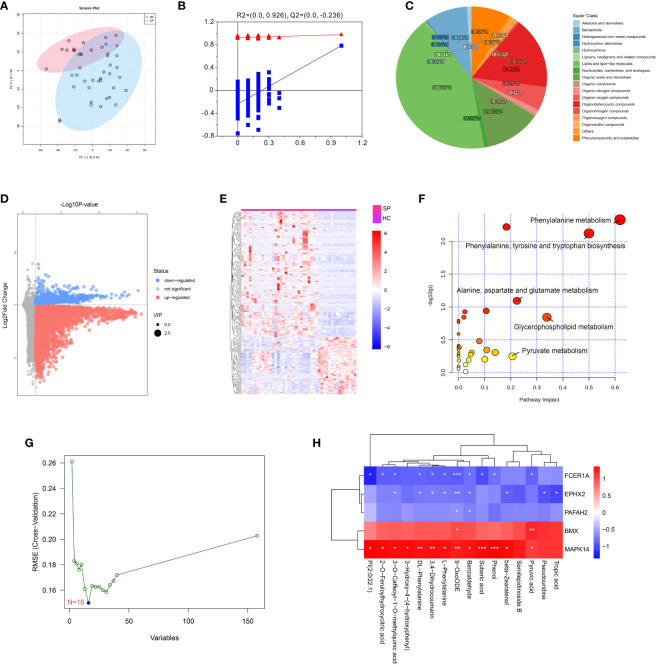
Metabolomics profiling of healthy control and sepsis patients. **(A)** Principal component analysis (PCA) scores plot for metabolomics analysis in sepsis and control. **(B)** OPLSDA plot. The ordinate represents the value of R^2^Y or Q^2^, the abscissa represents the degree of substitution retention, the red dot represents the R^2^Y value of the substitution test, the blue dot represents the Q^2^ value of the substitution test, and the two dashed lines represent the regression lines of R^2^Y and Q^2^, respectively. **(C)** Pie chart analysis of differentially-expressed metabolites categories. Each color represents a different category of substances. **(D)** Volcano plot, and **(E)** Heat map analyzed by TBtools showing the significantly changed metabolites in sepsis and control. **(F)** Bubble plot of enriched pathways of differentially-expressed metabolites. Each bubble represents a metabolic pathway, with the position and size of the bubble indicating the impact of the pathway in the topological analysis. The color of the bubble indicates the P-value, with redder colors indicating smaller P-value and more significant enrichment. **(G)** Support vector machine (SVM) for hub metabolites, the optimal variables were screened out based on the “e1071” package. **(H)** Person’s correlation analysis between hub metabolisms and identified hub genes. *: as compared with the Control group, *P <*0.05; **: as compared with the Control group, *P <*0.01; ***: as compared with the Control group, *P*<0.001.

To verify the effect of *MAPK14*, the only hub gene that was involved in all the states of monocyte differentiation in sepsis, metabolomics was performed by a UHPLC-MS system (the clinical information of was shown in **Supplementary Table 3**). The PCA score plot indicated that the two groups of samples have significant differentiation and were basically within the 95% confidence interval (**Figure 7A**). Then the orthogonal partial least squares-discriminant analysis (OPLS-DA) was established for pattern recognition of the two groups and to explore the differentially expressed metabolites (DEMs) (**Figure 7B**). A total of 449 DEMs were obtained, of which 309 were upregulated and 140 were downregulated in the sepsis group (VIP-value > 1, P-value < 0.05), and the super-classes of DEMs were shown in the pie chart (**Figures 7C–E**). DEMs were enriched in pathways, such as phenylalanine metabolism, phenylalanine, tyrosine and tryptophan biosynthesis, and pyruvate metabolism (**Figure 7F**). In addition, a total of 16 hub metabolites were identified by SVM (**Figure 7G**). The correlation between hub metabolites and identified hub genes were further analyzed by Pearson’s correlation analysis. Interestingly, these hub metabolites were all positively correlated with *MAPK14* and *BMX* and negatively correlated with *FCER1A*, *PAFAH2*, and *EPHX2* (**Figure 7H**).

**Figure 8:**
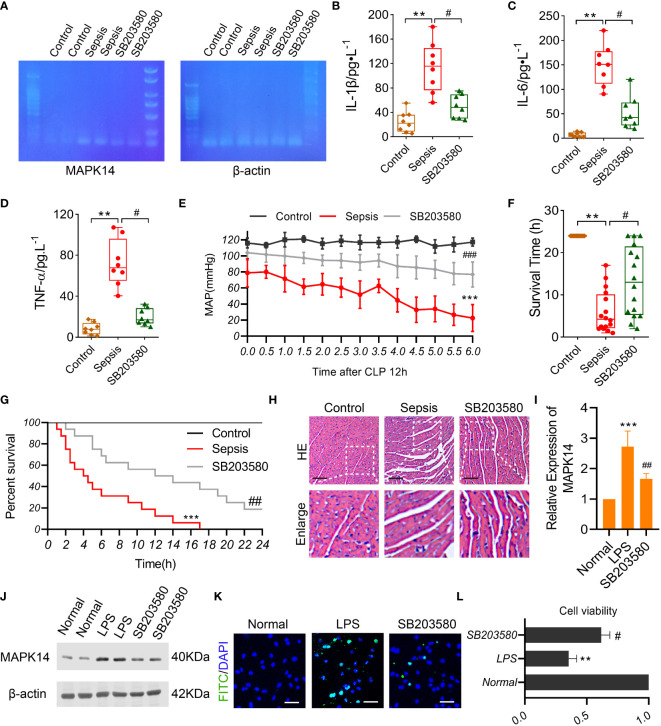
The role of MAPK14 in sepsis rats and LPS-induced cardiomyocytes. **(A)** RT-PCR detecting the inhibitory effectiveness of SB203580 on MAPK14 in heart tissues. ELISA showing the levels of **(B)** IL-1β, **(C)** IL-6, and **(D)** TNF-α between three groups. **(E)** MAP within 6h detecting starting at 12h after CLP. **(F)** The survival time and **(G)** rate of rats. **(H)** Representative HE staining images of myocardial fibers. **(I)** Quantification of Western blotting results of MAPK14. **(J)** Representative Western blotting images of MAPK14. **(K)** Representative TUNEL staining images of H9C2 cells. **(L)** CCK-8 detecting the cell viability of H9C2 cells. **: as compared with the Control or Normal group, *P*< 0.01; ***: as compared with the Control or Normal group, *P*< 0.001; ^#^: as compared with the Sepsis or LPS group, *P*< 0.05; ^##^: as compared with the Sepsis or LPS group, *P*< 0.01.

We established a sepsis rat model using CLP and administered them with a *MAPK14* antagonist SB203580. First, the inhibitory effectiveness of SB203580 on MAPK14 was confirmed in myocardial tissue of sepsis rats by gel electrophoresis (**Figure 8A**). The serum levels of pro-inflammatory cytokines IL-1β, IL-6, and TNF-α were significantly increased after sepsis, and down-regulated by inhibiting *MAPK14* to 56.7%, 64.8%, and 72.6% compared with the sepsis group, respectively (**Figures 8B–D**). The MAP declined from 12h after CLP, which was partly reversed by SB203580 treatment (**Figure 8E**). The survival rate of sepsis rat at 24h was 0, and the average survival time was 6.03 ± 4.96h, whereas the survival rate in the SB203580 group was 18.75% (3/16), and the average survival time was significantly extended to 13.28 ± 8.173h (**Figures 8F, G**).

Organ damage was an important cause of death in sepsis patients, and myocardial injury is one of the most severe complications of sepsis. To explore the effect of *MAPK14* antagonism on myocardial function in sepsis, we observed the structural changes of myocardial fibers in sepsis rats. The myocardial fibers were disorganized, and the space between fibers was widened after sepsis, and these changes were ameliorated by inhibiting *MAPK14* as the HE staining shown (**Figure 8H**). Then, we used a myocardial sepsis model *in vitro* by stimulating H9C2 cells with LPS (500 ng/ml) for 12h. The inhibitory effectiveness of SB203580 on *MAPK14* was verified by Western blot (**Figures 8I, J**). Next, TUNEL staining was performed to observe cell apoptosis in H9C2 cells. The TUNEL-positive cells were reduced in SB203580-treated H9C2 cells compared with LPS-induced ones (**Figure 8K**). In addition, the cell viability of H9C2 cells was decreased by 64.6% after being stimulated with LPS, while SB203580+LPS treatment increased the cell viability by 74.5% compared with the LPS stimulation (**Figure 8L**).

## Discussion

In the present study, we identified 508 DE-LMRGs between SP and HC and screened 5 lipid metabolism-related hub genes *MAPK14*, *EPHX2*, *BMX*, *FCER1A*, and *PAFAH2 via* machine learning algorithms. By analyzing the relationship between hub genes and immune-cell infiltration, we found an immunosuppressive and exhausted microenvironment in sepsis. Then, we investigated the immune cell landscape of these hub genes at the single-cell level by analyzing scRNA-seq data and identified the pivotal role of lipid metabolism in immune cells in sepsis. Finally, we validated the role of *MAPK14* in sepsis patients, rats, and LPS-induced cardiomyocytes.

Sepsis can advance to multiorgan system dysfunction that is caused by a dysregulated immune response to the infection and associated with high mortality and morbidity. Currently, there are still no effective treatment therapies to lower sepsis mortality due to the complex pathophysiology (28, 29). Although sepsis is fundamentally associated with inflammation, recent studies have reported that metabolism, especially lipid metabolism, plays a critical role in the pathogenesis and pathophysiology of sepsis (23, 30). Serum levels of the prostaglandins PGE_2_ and PGD_2_, two eicosanoid lipid mediators, are found to be elevated in patients with sepsis, accompanying with increased COX-2 activity (31). To explore the molecular mechanisms underlying sepsis, we analyzed public datasets and found that 508 DEGs were related to lipid metabolism, indicating that LMRGs might play critical roles in the sepsis pathophysiology.

Then the most relevant featured genes with sepsis were identified by WGCNA. Crucial DEGs were identified using LASSO logistic regression and RF. A total of 5 hub genes were found by taking the intersection of the results of multiple machine learning algorithms including LASSO, RF, and WGCNA, and they were all good candidates to predict sepsis validated by ROC curves. Based on these findings, these 5 hub genes could be used as prognostic indicators to predict the outcomes of patients with sepsis.

Platelet-activating factor acetyl-hydrolase type 2 (*PAFAH2*) is a hydrolytic enzyme that can remove oxidatively damaged lipids. *PAFAH2* has been found to repair oxidative-stress induced tissue injury and thus reduce related cell death (32). Therefore, *PAFAH2*, as a hub gene, may play a crucial role in lipid metabolism during sepsis and can predict the outcome of sepsis patients.

Belonged to the MAP kinase family, *MAPK14* act as an integration point for multiple biochemical signals, and participates in various cellular processes. *MAPK14* can be induced by many proinflammatory cytokines and is considered as a good predictor for sepsis, which is consistent with other bioinformatics analyses of sepsis (33–35).

*EPHX2* is a member of the epoxide hydrolase family. Mutations of *EPHX2* are associated with familial hypercholesterolemia, and *EPHX2* has also been proven to exacerbate acute vascular inflammatory responses (36), suggesting its role in lipid metabolism and inflammation. Therefore, *EPHX2* is a good candidate for predicting the prognosis of sepsis.

Bone marrow kinase on the X chromosome (*BMX*) encodes a non-receptor tyrosine kinase belonging to the Tec kinase family. *BMX* has been shown to attenuate endothelial permeability and vascular leakage during sepsis (37). In addition, *BMX* can regulate LPS-induced IL-6 and VEGF production and is involved in the phagocytosis of pathogens (38, 39). Thus, *BMX* may play a key role in the pathophysiology of sepsis as a hub gene.

*FCER1A*, an IgE receptor, is the initiator of the allergic response. In the present study, *FCER1A* was determined as a hub gene to the predict prognosis of sepsis, which was consistent with a previous study showing that *FCER1A* was identified as a potential diagnostic biomarker for sepsis (40, 41). In addition, *FCER1A* has been demonstrated to be associated with lipid metabolism and immune functions (42), supporting our findings.

Immune cells have different metabolic states and can preferentially utilize specific metabolites to perform corresponding functions (43). The alterations of these 5 hub genes in various immune cell types between SP and HC indicated that lipid metabolic changes played distinct roles in different immune cells. Besides, we found that some hub genes were positively correlated with the numbers of CD4^+^ T cells, CD8^+^ T cells, resting NK cells, and regulatory T cells, but negatively correlated with those of M1 macrophages, M2 macrophages, monocytes, and activated mast cells; whereas, the other hub genes showed the opposite trend, which provided novel insights into the regulation of subsets of immune cells. Metabolism reprogramming has been reported to be associated with immune cell infiltration (44). However, the exact metabolic pathways through which the 5 hub genes affecting the immune microenvironment of sepsis need to be further studied.

To further explore cellular and molecular features of each immune cell type involved in sepsis, we identified 7 cell clusters in PBMCs of sepsis patients using a public scRNA-seq dataset, and these immune cells were subclustered. First, the proportions of primary cell populations changed during disease progression. The proportions of monocytes and platelets increased while that of T cells and B cells decreased, suggesting phenotypic alterations of immune cells induced by sepsis. Among all these immune cells, we found that monocytes (DCs, non-classical monocytes, and classical monocytes) harbored the maximum number of cell-cell crosstalk with other neighboring cells in sepsis. All the 3 subsets of monocytes played crucial roles in the pathological process of sepsis. Accelerated differentiation of monocytes into DCs in sepsis patients were also reported (45, 46). Second, DEGs in each immune cell population was identified between HC and SP, indicating that sepsis could induce functional changes in different immune cells. Third, many immune cell subtypes were identified, and sepsis caused altered the expressions of 5 hub genes in almost all the subtypes, suggesting their pivotal roles in the immune system during sepsis.

The hub gene *MAPK14* might be critical in regulating the differentiation and function of monocytes as *MAPK14* was found to be involved in all the states of monocytes along the timeline during sepsis progression, and therefore, *MAPK14* might become a novel therapeutic target for sepsis. In addition to monocytes, the differentiation of T cells and B cells also played vital roles in the development of sepsis. However, the association between LMRGs and the differentiation of T and B cells remains unclear, which needs further investigation.

In this study, we also identified altered metabolite profiles of sepsis using metabolomics. The highest proportion of DEMs in sepsis is lipids and lipid-like molecules (41.9%). DEMs are mainly enriched in pyruvate metabolism. A total of 16 hub metabolites were found through the SVM algorithm, and their expressions were correlated with the expressions of the hub genes, indicating that the featured hub genes might have a pivotal role in the interplays among the dysregulated metabolites in sepsis. A recent study showed that the pathogenesis was influenced by metabolic homeostasis in sepsis (44). We also found that *MAPK14* was positively correlated with hub metabolites. In addition, the role of *MAPK14* in the development of sepsis was validated *in vivo* and *in vitro*. But the mechanism of the protective effect of *MAPK14* on monocyte differentiation and sepsis induced cardiac dysfunction was still unknown, and will be investigated in the future. Therefore, the novel sepsis biomarkers associated with metabolism, including *MAPK14* have the potential to develop targeted therapies for sepsis.

## Conclusions

In the present study, we identified 5 lipid metabolism-related hub genes (*MAPK14*, *EPHX2*, *BMX*, *FCER1A*, and *PAFAH2*) that have the possibility of diagnostic and therapeutic in patients with sepsis by machine learning analysis. The single-cell RNA landscape revealed that LMRGs might play pivotal roles in the immune system during sepsis. The protective effect of inhibiting *MAPK14* on sepsis indicated that these lipid-metabolic hub genes might have great potential in prognosis prediction and precise treatment for sepsis patients.

## Data availability statement

The datasets presented in this study can be found in online repositories. The names of the repository/repositories and accession number(s) can be found below: CNP0004111(CNGBdb).

## Ethics statement

The study was registered and approved by the Chinese Clinical Trial Registry (ChiCTR2200055772). The patients/participants provided their written informed consent to participate in this study. The ethics and the protocols for the animal experiments were approved by the Ethical Committee of Army Medical University.

## Author contributions

HS, TL, YH, and LL conceived and designed the study. HS, LT, and YW analyzed the data. HS, TL, and YW drafted the manuscript. TL, LL and YH revised the manuscript. TL, LL, and HS acquired the financial support. All authors performed the experimental procedures. All authors contributed to the article and approved the submitted version.

## Supplementary material

Supplementary Figure 1
**(A)** UMAP plots of clusters in each sample. **(B)** DEGs between HC and SP in T cells, B cells, and monocytes.

Supplementary Figure 2
**(A)** Subclusters and populations of Platelets. **(B)** Subclusters and populations of Neutrophils.

## References

[1] Bauer M Gerlach H Vogelmann T Preissing F Stiefel J Adam D. Mortality in sepsis and septic shock in Europe, north America and Australia between 2009 and 2019- results from a systematic review and meta-analysis. Crit Care (2020) 24(1):239. doi: 10.1186/s13054-020-02950-2 32430052 PMC7236499

[2] Fang M Zou T Yang X Zhang Z Cao P Han J. Discovery of novel pterostilbene derivatives that might treat sepsis by attenuating oxidative stress and inflammation through modulation of MAPKs/NF-κB signaling pathways. Antioxidants (2021) 10(9):1333. doi: 10.3390/antiox10091333 34572964 PMC8470242

[3] Rudd KE Kissoon N Limmathurotsakul D Bory S Mutahunga B Seymour CW. The global burden of sepsis: barriers and potential solutions. Crit Care (2018) 22(1):232. doi: 10.1186/s13054-018-2157-z 30243300 PMC6151187

[4] Rhodes A Evans LE Alhazzani W Levy MM Antonelli M Ferrer R. Surviving sepsis campaign: international guidelines for management of sepsis and septic shock: 2016. Crit Care Med (2017) 45(3):486–552. doi: 10.1097/CCM.0000000000002255 28098591

[5] Oberhoffer M Vogelsang H Russwurm S Hartung T Reinhart K. Outcome prediction by traditional and new markers of inflammation in patients with sepsis. Clin Chem Lab Med (1999) 37(3):363–8. doi: 10.1515/CCLM.1999.060 10353484

[6] Phua J Koay ES Lee KH. Lactate, procalcitonin, and amino-terminal pro-b-type natriuretic peptide versus cytokine measurements and clinical severity scores for prognostication in septic shock. Shock (2008) 29(3):328–33. doi: 10.1097/SHK.0b013e318150716b 18277855

[7] Hamasaki MY Barbeiro HV de Souza HP Machado MC da Silva FP. sRAGE in septic shock: a potential biomarker of mortality. Rev Bras Ter Intensiva (2014) 26(4):392–6. doi: 10.5935/0103-507X.20140060 PMC4304468 25607269

[8] da Gomes Cunha DéboraM da Silva GG Hamasaki MY. New biomarkers of sepsis with clinical relevance. Clin Manage Shock - Sci Art Physiol Restor (2019). doi: 10.5772/intechopen.82156

[9] Michie HR. Metabolism of sepsis and multiple organ failure. World J Surg (1996) 20(4):460–4. doi: 10.1007/s002689900072 8662135

[10] Singer M. The role of mitochondrial dysfunction in sepsis-induced multi-organ failure. Virulence (2014) 5(1):66–72. doi: 10.4161/viru.26907 24185508 PMC3916385

[11] Lewis AJ Billiar TR Rosengart MR. Biology and metabolism of sepsis: innate immunity, bioenergetics, and autophagy. Surg Infect (Larchmt) (2016) 17(3):286–93. doi: 10.1089/sur.2015.262 PMC4876546 27093228

[12] Amunugama K Pike DP Ford DA. The lipid biology of sepsis. J Lipid Res (2021) 62:100090–0. doi: 10.1016/j.jlr.2021.100090 PMC8243525 34087197

[13] Schork NJ. Artificial intelligence and personalized medicine. Cancer Treat Res (2019) 178:265–83. doi: 10.1007/978-3-030-16391-4_11 PMC7580505 31209850

[14] Wang D Li J Sun Y Ding X Zhang X Liu S. A machine learning model for accurate prediction of sepsis in ICU patients. Front Public Health (2021) 9:754348. doi: 10.3389/fpubh.2021.754348 34722452 PMC8553999

[15] Deng H-F Sun M-W Wang Y Zeng J Yuan T Li T. Evaluating machine learning models for sepsis prediction: a systematic review of methodologies. iScience (2022) 25(1):103651. doi: 10.1016/j.isci.2021.103651 35028534 PMC8741489

[16] Pinu FR Beale DJ Paten AM Kouremenos K Swarup S Schirra HJ. Systems biology and multi-omics integration: viewpoints from the metabolomics research community. Metabolites (2019) 9(4):76. doi: 10.3390/metabo9040076 31003499 PMC6523452

[17] Reyes M Filbin MR Bhattacharyya RP Billman K Eisenhaure T Hung DT. An immune-cell signature of bacterial sepsis. Nat Med (2020) 26(3):333–40. doi: 10.1038/s41591-020-0752-4 PMC7235950 32066974

[18] Kominsky DJ Campbell EL Colgan SP. Metabolic shifts in immunity and inflammation. J Immunol (2010) 184(8):4062–8. doi: 10.4049/jimmunol.0903002 20368286 PMC4077461

[19] Kumar V. Immunometabolism: another road to sepsis and its therapeutic targeting. Inflammation (2019) 42(3):765–88. doi: 10.1007/s10753-018-0939-8 30506105

[20] Delano MJ Ward PA. Sepsis-induced immune dysfunction: can immune therapies reduce mortality? J Clin Invest (2016) 126(1):23–31. doi: 10.1172/jci82224 26727230 PMC4701539

[21] Hohlstein P Gussen H Bartneck M Warzecha KT Roderburg C Buendgens L. Prognostic relevance of altered lymphocyte subpopulations in critical illness and sepsis. J Clin Med (2019) 8(3):353. doi: 10.3390/jcm8030353 30871101 PMC6463123

[22] Haque A Engel J Teichmann SA Lönnberg T. A practical guide to single-cell RNA-sequencing for biomedical research and clinical applications. Genome Med (2017) 9(1):75. doi: 10.1186/s13073-017-0467-4 28821273 PMC5561556

[23] She H Tan L Zhou Y Zhu Y Ma C Wu Y. The landscape of featured metabolism-related genes and imbalanced immune cell subsets in sepsis. Front Genet (2022) 13:821275. doi: 10.3389/fgene.2022.821275 35265105 PMC8901109

[24] Qiu X Li J Bonenfant J Jaroszewski L Mittal A Klein W. Dynamic changes in human single-cell transcriptional signatures during fatal sepsis. J Leukoc Biol. (2021) 110(6):1253–68. 10.1002/JLB.5MA0721-825R PMC8629881 34558746

[25] Stuart T Butler A Hoffman P Hafemeister C Papalexi E Mauck WM. Comprehensive integration of single-cell data. Cell (2019) 177(7):1888–1902.e1821. doi: 10.1016/j.cell.2019.05.031 31178118 PMC6687398

[26] Qiu X Hill A Packer J Lin D Ma YA Trapnell C. Single-cell mRNA quantification and differential analysis with census. Nat Methods (2017) 14(3):309–15. doi: 10.1038/nmeth.4150 PMC5330805 28114287

[27] Fang Y Wu Y Liu L Wang H. The four key genes participated in and maintained atrial fibrillation process *via* reprogramming lipid metabolism in AF patients. Front Genet (2022) 13:821754. doi: 10.3389/fgene.2022.821754 35669184 PMC9163572

[28] Plevin R Callcut R. Update in sepsis guidelines: what is really new? Trauma Surg acute Care Open (2017) 2(1):e000088–e000088. doi: 10.1136/tsaco-2017-000088 29766091 PMC5877904

[29] Martin-Loeches I Guia MC Vallecoccia MS Suarez D Ibarz M Irazabal M. Risk factors for mortality in elderly and very elderly critically ill patients with sepsis: a prospective, observational, multicenter cohort study. Ann Intensive Care (2019) 9(1):26. doi: 10.1186/s13613-019-0495-x 30715638 PMC6362175

[30] Englert JA Rogers AJ. Metabolism, metabolomics, and nutritional support of patients with sepsis. Clinics chest Med (2016) 37(2):321–31. doi: 10.1016/j.ccm.2016.01.011 PMC5084839 27229648

[31] Ahmad NS Tan TL Arifin KT Ngah WZW Yusof YAM. High sPLA2-IIA level is associated with eicosanoid metabolism in patients with bacterial sepsis syndrome. PloS One (2020) 15(3):e0230285. doi: 10.1371/journal.pone.0230285 32160261 PMC7065791

[32] Adibekian A Hsu KL Speers AE Monillas ES Brown SJ Spicer T. Optimization and characterization of a triazole urea inhibitor for platelet-activating factor acetylhydrolase type 2 (PAFAH2). In: Probe reports from the NIH molecular libraries program. Bethesda (MD: National Center for Biotechnology Information (US (2010). 23658960

[33] Zeng X Feng J Yang Y Zhao R Yu Q. Screening of key genes of sepsis and septic shock using bioinformatics analysis. J Inflammation Res (2021) 14:829–41. doi: 10.2147/jir.s301663 PMC7962593 33737824

[34] Zhang X Cui Y Ding X Liu S Han B Duan X. Analysis of mRNA−lncRNA and mRNA−lncRNA-pathway co−expression networks based on WGCNA in developing pediatric sepsis. Bioengineered (2021) 12(1):1457–70. doi: 10.1080/21655979.2021.1908029 PMC8806204 33949285

[35] Zhou X Wang Y. Constructing a 10-core genes panel for diagnosis of pediatric sepsis. J Clin Lab Anal (2021) 35(3):e23680. doi: 10.1002/jcla.23680 33274532 PMC7958006

[36] Deng Y Edin ML Theken KN Schuck RN Flake GP Kannon MA. Endothelial CYP epoxygenase overexpression and soluble epoxide hydrolase disruption attenuate acute vascular inflammatory responses in mice. FASEB J (2011) 25(2):703–13. doi: 10.1096/fj.10-171488 PMC3023387 21059750

[37] Li Z Yin M Zhang H Ni W Pierce RW Zhou HJ. BMX Represses thrombin-PAR1-Mediated endothelial permeability and vascular leakage during early sepsis. Circ Res (2020) 126(4):471–85. doi: 10.1161/circresaha.119.315769 PMC7035171 31910739

[38] Palmer CD Mutch BE Page TH Horwood NJ Foxwell BM. Bmx regulates LPS-induced IL-6 and VEGF production *via* mRNA stability in rheumatoid synovial fibroblasts. Biochem Biophys Res Commun (2008) 370(4):599–602. doi: 10.1016/j.bbrc.2008.03.142 18402776

[39] Koprulu AD Ellmeier W. The role of tec family kinases in mononuclear phagocytes. Crit Rev Immunol (2009) 29(4):317–33. doi: 10.1615/critrevimmunol.v29.i4.30 19673686

[40] Xue L Wu P Zhao X Jin X Wang J Shi Y. Using immune-related lncRNA signature for prognosis and response to immunotherapy in cutaneous melanoma. Int J Gen Med (2021) 14:6463–75. doi: 10.2147/IJGM.S335266 PMC8518697 34675614

[41] Fan Y Han Q Li J Ye G Zhang X Xu T. Revealing potential diagnostic gene biomarkers of septic shock based on machine learning analysis. BMC Infect Dis (2022) 22(1):65. doi: 10.1186/s12879-022-07056-4 35045818 PMC8772133

[42] Massot-Cladera M Franch À. Castell M Pérez-Cano FJ. Cocoa polyphenols and fiber modify colonic gene expression in rats. Eur J Nutr (2017) 56(5):1871–85. doi: 10.1007/s00394-016-1230-0 PMC5534200 27256297

[43] Ganeshan K Chawla A. Metabolic regulation of immune responses. Annu Rev Immunol (2014) 32:609–34. doi: 10.1146/annurev-immunol-032713-120236 PMC5800786 24655299

[44] Van Wyngene L Vandewalle J Libert C. Reprogramming of basic metabolic pathways in microbial sepsis: therapeutic targets at last? EMBO Mol Med (2018) 10(8):e8712. doi: 10.15252/emmm.201708712 29976786 PMC6079534

[45] Hotchkiss RS Tinsley KW Swanson PE Grayson MH Osborne DF Wagner TH. Depletion of dendritic cells, but not macrophages, in patients with sepsis. J Immunol (2002) 168(5):2493–500. doi: 10.4049/jimmunol.168.5.2493 11859143

[46] Faivre V Lukaszewicz AC Alves A Charron D Payen D Haziot A. Accelerated *in vitro* differentiation of blood monocytes into dendritic cells in human sepsis. Clin Exp Immunol (2007) 147(3):426–39. doi: 10.1111/j.1365-2249.2006.03287.x PMC1810505 17302891

