# Corrigendum: Integrative single-cell RNA sequencing and metabolomics decipher the imbalanced lipid-metabolism in maladaptive immune responses during sepsis

**DOI:** 10.3389/fimmu.2024.1418495

**Published:** 2024-04-22

**Authors:** Han She, Lei Tan, Yi Wang, Yuanlin Du, Yuanqun Zhou, Jun Zhang, Yunxia Du, Ningke Guo, Zhengbin Wu, Qinghui Li, Daiqin Bao, Qingxiang Mao, Yi Hu, Liangming Liu, Tao Li

**Affiliations:** ^1^ State Key Laboratory of Trauma, Burns and Combined Injury, Shock and Transfusion Department, Daping Hospital, Army Medical University, Chongqing, China; ^2^ Department of Anesthesiology, Daping Hospital, Army Medical University, Chongqing, China; ^3^ Department of Intensive Care Unit, Daping Hospital, Army Medical University, Chongqing, China

**Keywords:** sepsis, lipid-metabolism, machine learning algorithm, single-cell RNA sequencing, metabolomics

**Missing Citation**.

In the published article the reference Qiu X, Li J, Bonenfant J, Jaroszewski L, et al. Dynamic changes in human single-cell transcriptional signatures during fatal sepsis. J Leukoc Biol. 2021 Dec;110(6):1253-1268. PMID: 34558746. was not cited in the article. The citation has now been inserted in **Materials and methods section**; *Single cell RNA-seq analysis*, as followed:

“The scRNA-seq dataset GSE167363 [24] (included 5 sepsis patients and 2 healthy controls) was analyzed in this study”.

The authors apologize for this error and state that this does not change the scientific conclusions of the article in any way. The original article has been updated.

