# Supplementary material for: Integrative single-cell RNA sequencing and metabolomics decipher the imbalanced lipid-metabolism in maladaptive immune responses during sepsis
Source: Front Immunol. 2023 Apr 27;14:1181697. doi: 10.3389/fimmu.2023.1181697 (PMC10172510; doi:10.3389/fimmu.2023.1181697)
Supplement: Supplementary file 1 [file Table_1.docx]

**S****upplementary Table 1:** The inclusion and exclusion criteria of sepsis patients.

| Inclusion criteria | 1. Diagnosed as sepsis. 2. The patient informed consent and participated voluntarily. 3. 18-80 years old (including 80 years old). 4. Register general medical conditions. |
| --- | --- |
| Exclusion criteria | 1. Age>80 years or ≤18 years. 2. Previous history of chronic heart disease, liver and kidney diseases. 3. Pregnant or lactating women. 4. Hyperlipidemia, diabetes, or other metabolic diseases. 5. Complicated with cardiovascular, liver, kidney and hematopoietic system and other serious primary diseases. 6. Combined with psychiatric diseases. 7. Long-term use of sedative drugs or alcohol. 8. Complicated with tumor or immune deficiency and other diseases that have a greater impact on immunity. 9. Patient or family refusal to be involved in the study. |
