# Supplementary material for: Integrative single-cell RNA sequencing and metabolomics decipher the imbalanced lipid-metabolism in maladaptive immune responses during sepsis
Source: Front Immunol. 2023 Apr 27;14:1181697. doi: 10.3389/fimmu.2023.1181697 (PMC10172510; doi:10.3389/fimmu.2023.1181697)
Supplement: Supplementary file 2 [file Table_2.docx]

**Supplementary Table 2: qRT-PCR primer sequences of hub genes.**

| **Genes** | **Forward primers** | **Reverse primers** |
| --- | --- | --- |
| *MAPK14* | *GAACTTCGCAAATGTATTTATTGGT* | *CGAGTCCAAAACCAGCATCT* |
| *EPHX2* | *CTGTAAGGCGTTGGGAAGGA* | *CGATGTGTCCCCTTTTCAGGA* |
| *BMX* | *TTATGACTCCCAGCCAACCG* | *TACTGCGGGCAATGTCTTCC* |
| *FCER1A* | *AACGTGATGCAAGAGTGGCT* | *TTCTTCCAGCTACGGCATCT* |
| *PAFAH2* | *CTATAGCCCTGGACGCTTGG* | *TCACGATCCTGGATTGCTCG* |
| *β-Actin* | *CATGTACGTTGCTATCCAGGC* | *CTCCTTAATGTCACGCACGAT* |
