# Supplementary figures and images for: Integrative single-cell RNA sequencing and metabolomics decipher the imbalanced lipid-metabolism in maladaptive immune responses during sepsis
Source: Front Immunol. 2023 Apr 27;14:1181697. doi: 10.3389/fimmu.2023.1181697 (PMC10172510; doi:10.3389/fimmu.2023.1181697)

Supplementary Figure1

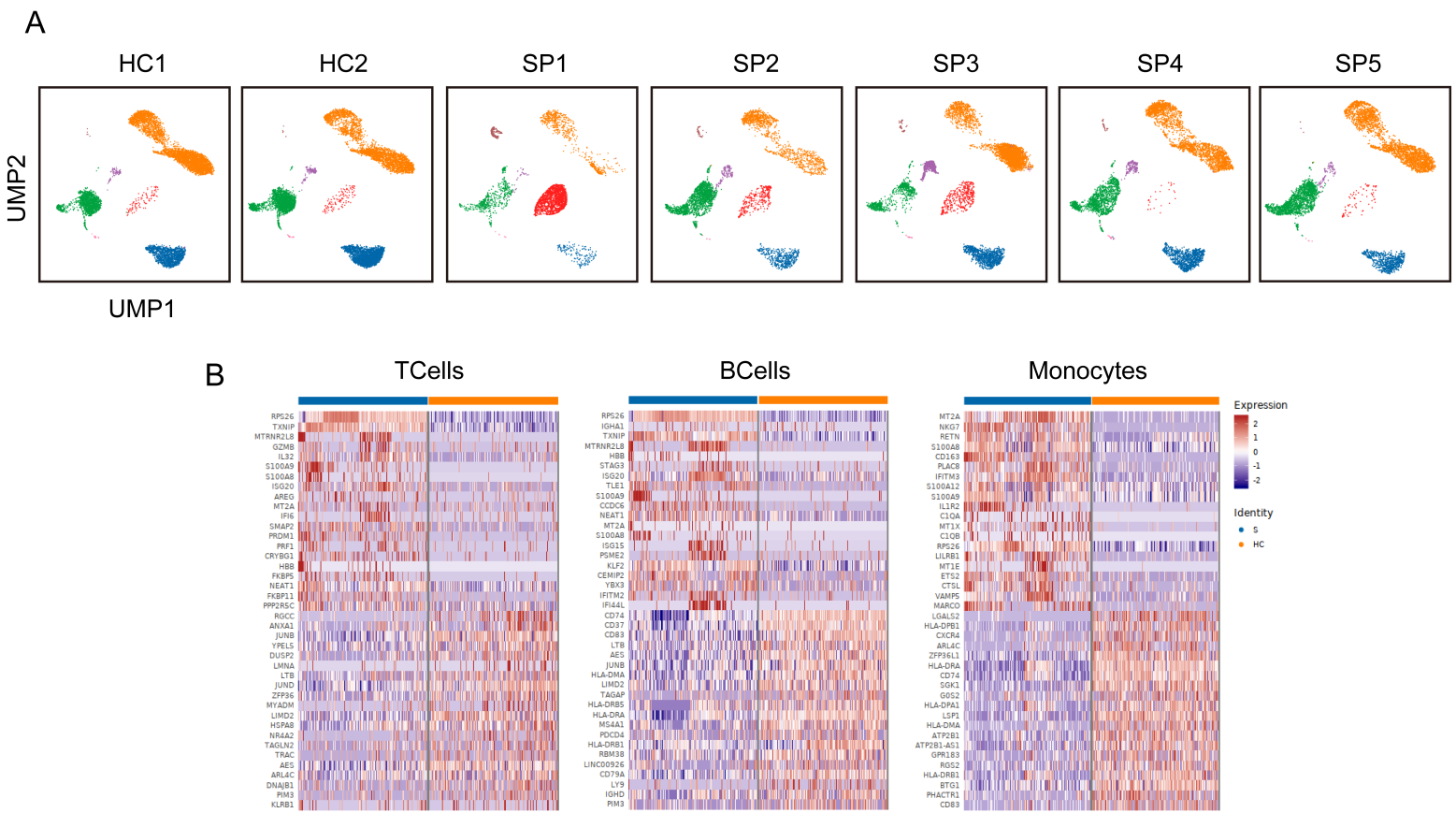

Supplement: Supplementary Figure 1 — (A) UMAP plots of clusters in each sample. (B) DEGs between HC and SP in T cells, B cells, and monocytes. [file DataSheet_1.pdf]

Supplementary Figure2

A

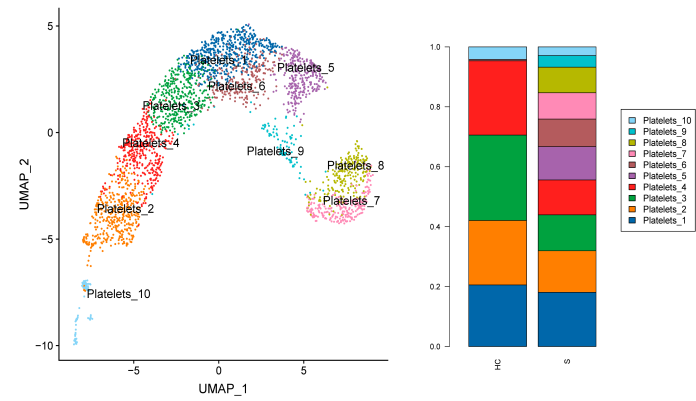

B

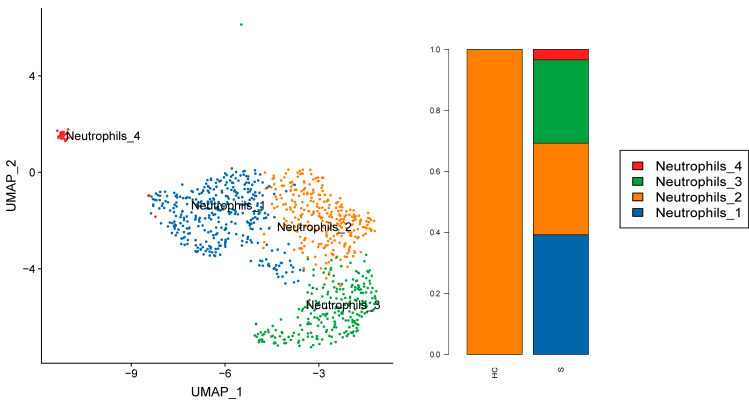

Supplement: Supplementary Figure 2 — (A) Subclusters and populations of Platelets. (B) Subclusters and populations of Neutrophils. [file DataSheet_2.pdf]
